# PAIP1 is a novel oncogene in human hepatocellular carcinoma

**DOI:** 10.1007/s12672-022-00530-0

**Published:** 2022-11-27

**Authors:** Nuobei Zhang, Xin Chen

**Affiliations:** 1 Department of Gastroenterology, The Second Affiliated Hospital of Nanchang University, No. 1 Minde Road, Nanchang, 330006 China; 2 Department of Nuclear Medicine, The Second Affiliated Hospital of Nanchang University, Nanchang, China

**Keywords:** Poly(a) interacting protein 1, Hepatocellular carcinoma, Oncogene, siRNA, Bioinformatics

## Abstract

**Background:**

Poly(A)-binding protein interacting protein 1 (PAIP1) is a translational initiation regulatory factor that has been reported as oncogene in multiple malignant diseases. However, its role in hepatocellular carcinoma (HCC) and the potential mechanisms have not been explored.

**Methods:**

PAIP1 expression level in HCC cell lines were detected by real-time quantitative PCR and western blotting. The proliferation and colony formation of HCC cell lines were detected by MTT and colony formation assay. The apoptosis and cell cycle were detected by flow cytometry. The volume and growth rate of the xenograft tumors were observed. The potential mechanism of PAIP1 was analyzed by miRNA Microarray Analysis and TargetScan analysis.

**Results:**

PAIP1 is significantly upregulated in HCC cell lines. PAIP1 knockdown dramatically inhibits cell proliferation and colony formation, induces apoptosis and alters the cell cycle distribution by increasing the G2/M cell percentage. Moreover, PAIP1 knockdown significantly reduces tumorigenesis in a murine transplantation model. Bioinformatics and immunoblotting analysis reveal that PAIP1 knockdown dysregulates cyclin D pathway-related proteins.

**Conclusion:**

PAIP1 plays an oncogenic role in hepatocellular carcinoma.

**Supplementary Information:**

The online version contains supplementary material available at 10.1007/s12672-022-00530-0.

## Introduction

Primary liver cancer is the sixth most common malignancy, with hepatocellular carcinoma (HCC) accounting for 80–90% of primary liver cancer cases [1, 2]. Notably, HCC is the second most common cause of cancer mortality following lung cancer [1, 2]. Moreover, researchers estimate an approximately 30% increase in HCC cases over the next decade in most regions worldwide. Unfortunately, 25–70% of HCC patients are diagnosed with incurable advanced-stage disease [1]. These patients have limited therapeutic options, as conventional chemotherapeutic regimens provide minimal clinical benefit [1].

To address this challenge, there is a substantial need for novel, effective molecular-based therapeutics against HCC. To the end, the carcinogenesis of HCC has been connected to various defects in the regulation of translational initiation [3–5]. During translational initiation, poly(A)-binding protein (PABP) and eukaryotic translational initiation factor 3 (eIF3) interact cooperatively with the mRNA’s 3’ poly(A) for ribosomal recruitment [6]. One key regulatory factor, PABP interacting protein 1 (PAIP1), directly interacts with PABP and eIF3 to enhance ribosomal recruitment by stabilizing interaction of PABP with eIF4G [6]. Recent work has revealed that oncogenic mTOR-S6K signaling promotes the pro-translational PAIP1-eIF3 interaction [7, 8], while the ubiquitin ligase WWP2 targets PAIP1 for proteasomal degradation, thereby suppressing translation [9]. These findings suggest that PAIP1 expression is necessary to maintain normal translational initiation but PAIP1 hyper-activation may contribute to carcinogenesis.

Of clinical relevance, PAIP1 has been implicated in several human malignancies, including bladder cancer, cervical cancer, and breast cancer [10–14]. Despite the fact that changes in PAIP1 expression have been implicated in several carcinomas, its exact role in human HCC remains unknown. In this study, we investigated the potential role of PAIP1 in human HCC based on in vitro and in vivo experiments. Furthermore, the mechanism of PAIP1 in HCC was explored.

## Materials and methods

### 1Cell culture

The human HCC cell lines HEPG2, HUH-7, and HEP3B were obtained from American Type Culture Collection (ATCC, Manassas, VA, USA), and the HCC cell line SMMC-7721 and the normal human hepatocyte cell line L02 were obtained from the Cell Research Institute (Chinese Academy of Sciences, Shanghai, China). Each cell line was independently maintained in Dulbecco’s Modified Eagle’s Medium (DMEM; Gibco, Carlsbad, CA, USA) supplemented with 10% fetal bovine serum (FBS, Gibco) and 100 U/ml of penicillin–streptomycin (Gibco). Cultured cells were maintained at 37 °C in a humidified incubator with 5% CO_2_.

### Lentiviral PAIP1 knockdown

Lentiviral PAIP1 knockdown in HCC cells was performed as previously described with minor modifications [15]. Small interfering RNAs (siRNAs) duplexes for *PAIP1* were synthesized by Guangzhou Ribobio Co. (Guangzhou, China) as follows (where dT is deoxyribosylthymine): 5’-CAU GUC GGA CGG UUU CGA UdTdT-3’ and 5’-GCU GCA AAA GGG GAU GAA GdTdT-3’ [16]. The inverted 4E-T siRNA was employed as a negative control siRNA (NC-siRNA) [16]. The siRNA sequences were independently cloned into pLKO.3G containing an eGFP marker, a gift from Christophe Benoist & Diane Mathis (Addgene plasmid #14,748). Using Lipofectamine 2000 (Invitrogen), HEK293T cells were co-transfected with either pLKO.3G-PAIP1-siRNA or pLKO.3G-NC-siRNA along with the packaging plasmid psPAX2 and the envelope plasmid pMD2.G at a 4:3:2 ratio. The viral supernatants were harvested after 48 h, and viral titers were quantified. The lentiviral supernatants were then added to the target HCC cell lines at an MOI of 10 with Enhanced Infection Solution (ENi.S) and polybrene (6 μg/ml, Sigma). Lentiviral infection efficiency was assessed by GFP microscopy 24 h after infection, where we found a lentiviral infection rate exceeding 80% (Additional file 1: Fig. S1).

### Real-time quantitative polymerase chain reaction (RT-PCR)

Total RNA was extracted from human HCC cell lines using the RNeasy Mini Kit (Qiagen, Valencia, CA, USA). To measure mRNA levels, total RNA was reverse transcribed into cDNA using the Sensiscript RT Kit (Qiagen) in accordance with the manufacturer’s instructions. RT-PCR was performed using SYBR Green PCR Master Mix (Roche Applied Science, Indianapolis, IN, USA) on an ABI Prism 7900 Sequence Detection System (Applied Biosystems, Foster City, CA, USA). The primers for amplification of PAIP1 were as follows: (forward primer) 5’-TCA GGA TCC GCT AAG CCC CAG GTG GTT-3’ and (reverse primer) 5’-GGC TCG AGT TAC TGT TTT CGC TTA CG-3’ [16]. The expression level of glyceraldehyde 3-phosphate dehydrogenase (GAPDH) was used as the endogenous control for sample normalization (forward primer: 5’-CCA CCC ATG GCA AAT TCC ATG GCA-3’; reverse primer, 5’-TCT AGA CGG CAG GTC AGG TCC ACC-3’). Standard thermocycler conditions were employed for data collection, and fold changes were calculated by means of relative quantification (2^−ΔΔCT^) as previously described by Livak et al. [17].

### Western blotting

Cell lysis was performed using a lysis buffer (Sigma, St. Louis, MO, USA) with a protease inhibitor (Promega, Madison, WI, USA). Cell lysates containing equivalent protein amounts were separated by sodium dodecyl sulphate polyacrylamide gel electrophoresis (SDS-PAGE, 10%) and transferred onto polyvinylidene fluoride membranes (Millipore, Billerica, MA, USA) via a Mini TransBlot system (Bio-Rad, Hercules, CA, USA). The blots were blocked for 1 h in 5% milk prepared in TBST, and blocked membranes were subsequently incubated at 4 °C overnight with the following antibodies (Cell Signaling Technology, Danvers, MA, USA unless otherwise specified): anti-PAIP1 (diluted 1:5000; cat#ab175211, Abcam, Cambridge, UK), anti-cyclin-dependent kinase 6 (CDK6; diluted 1:200, cat#3136), anti-cyclin D1 (CCND1; diluted 1:500, cat#2978), anti-cyclin D2 (CCND2; diluted 1:500, cat#3741), anti-mouse double minute 2 homolog (MDM2; diluted 1:200, cat#86,934), and anti-GAPDH (diluted 1:1000, cat#2118). The next day, membranes washed and incubated with species-specific secondary antibodies conjugated to horseradish peroxidase for 1 h at room temperature (diluted 1:2000), followed by several washes with TBST to remove unbound antibody. Finally, enhanced chemiluminescence (Pierce, Rockford, IL, USA) was used to visualize the protein expression levels in accordance with the manufacturer's instructions. GAPDH was used as the internal control. Densitometric analysis was performed with Image-J (NIH, Bethesda, MD, USA).

### Cell proliferation assays

For the Cellomics assay, cells were cultured in a 96-well plate for 24 h after lentiviral infection prior to experimentation. Green fluorescence protein (GFP)-expressing cells were quantified once daily for five days using a Cellomics ArrayScan system (Thermo Scientific, Waltham, MA, USA). For the 3-(4, 5-dimethylthiazol-2-yl)-2, 5-diphenyltetrazolium bromide (MTT) assay, cells were cultured in a 96-well plate for 24 h after lentiviral infection prior to experimentation. At the indicated time points, medium was replaced with fresh medium supplemented with MTT (0.5 mg/ml) (Sigma). Absorbance was measured at a wavelength of 490 nm (OD490).

### Apoptosis assay

Apoptosis was examined using an Apoptosis Detection kit that specifically comprised a FITC-labeled Annexin V/propidium iodide (PI) method (Invitrogen). In brief, cells were infected with the PAIP1-siRNA or the NC-siRNA and cultured for 48 h. Then cells were collected and washed with cold PBS followed by stained with dye buffer containing 10 μl of FITC-A and 10 μl of PI for 15 min at room temperature. Then the stained cells were analyzed by a FACSCalibur (BD Biosciences, San Jose, CA, USA) and analyzed using FLOWJO software (Tree Star, Ashland, OR, USA).

### Colony formation assay

Cells were infected with the PAIP1-siRNA or the NC-siRNA and cultured for 24 h. The cells were then collected and plated in six-well plates at a density of 5 × 10^2^ cells per well. After 10 days, cells were washed with phosphate-buffered saline, followed by fixation with methanol/acetic acid (3:1; v:v), and finally stained with Giemsa [18]. The number of colonies were counted under a microscope [19].

### Cell cycle analysis

For cell cycle analysis, cells were first resuspended in ice-cold phosphate buffered saline. To measure DNA content, cells were stained with PI solution (50 µg/ml PI, 100 µg/ml RNase A, 0.05% Triton X-100 in NaCl/P_i_) and then incubated in the dark at 37 °C for 30 min. DNA content was observed by flow cytometry using a FACSCalibur (BD Biosciences, San Jose, CA, USA) and analyzed using FLOWJO software (Tree Star, Ashland, OR, USA).

### Subcutaneous xenograft tumor model

Female BALB/c nude mice (4–6 weeks of age) were purchased from Shanghai SLAC Laboratory Animal Co. (Shanghai, China). PAIP-siRNA-infected SMMC-7721 cells (5 × 10^6^; KD group) or NC-siRNA-infected SMMC-7721 cells (5 × 10^6^; NC group) were injected subcutaneously (s.c.) into nude mice via their dorsal flanks. Each group contained 10 mice. Tumor volumes (V) were measured daily by calipers and were calculated by the following formula: V = 1/2 × S^2^ × L, where S and L are the shortest and longest diameter of the tumor, respectively. Mice were sacrificed four weeks after injection, and final tumor weights were measured.

### miRNA microarray analysis

Total RNA was extracted from the foregoing SMMC-7721 xenograft tumors using the RNeasy Mini Kit (Qiagen, Valencia, CA, USA). RNA (2 µg) was labelled and hybridized to a Genechip miRNA v.2.0 microarray (Affymetrix, CA, USA). Then, the raw data were quartile-normalized with the miRNAQC software (Affymetrix) prior to exporting the intensity data into Genespring 7.2 software (Agilent, CA, USA). In order to perform the unsupervised cluster analysis according to the Pearson correlation algorithm, the non-hybridized probes were removed by filtering probes with the flag = “TRUE” function. Thereafter, an analysis of variance (ANOVA) was applied to identify differentially-expressed mature human miRNAs between the NC and KD samples. *P*-values were then corrected with the Benjamini-Hogberg method. Processing of each sample was repeated in triplicate.

### KEGG pathway analysis

TargetScan software (http://www.targetscan.org) was then applied to predict putative target genes for the differentially-expressed mature human miRNAs between the NC and KD samples. The Kyoto Encyclopedia of Genes and Genomes (KEGG) pathway database was then used to identify the significantly-enriched biological pathways for these putative target genes. *P*-values (via two-sided Fisher’s exact testing) and *q*-values were calculated to select significantly-enriched pathways and control for the false discovery rate (FDR), respectively [20].

### Statistical analysis

Data are expressed as mean ± standard deviation (SD). Differences between groups were analyzed using either the two-tailed paired or unpaired Student’s *t*-test. A *P*-value of less than 0.05 was considered statistically significant.

## Results

### PAIP1 expression was upregulated in human HCC cell lines

**Fig.1:**
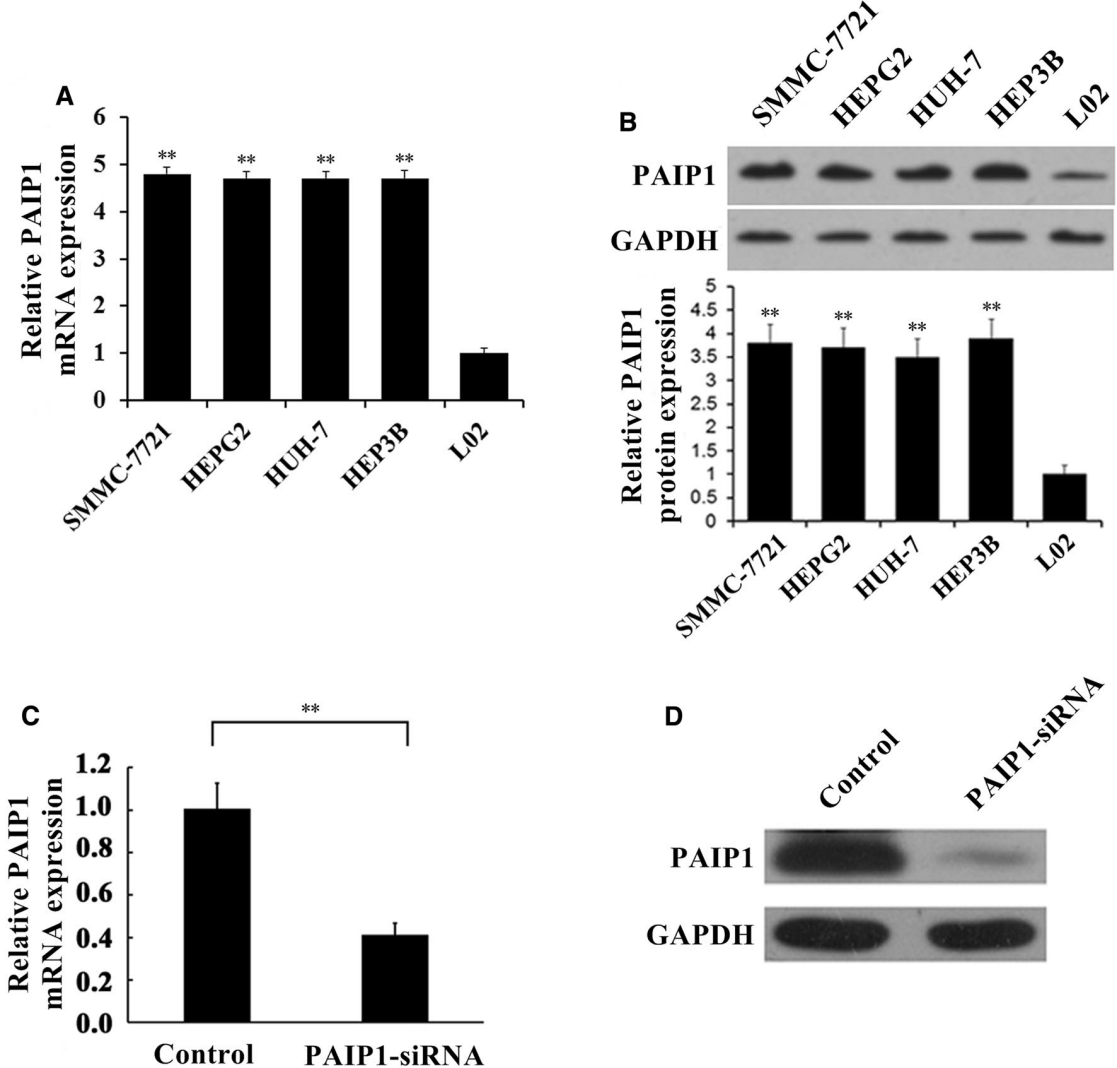
PAIP1 Upregulation in HCC Cell Lines and PAIP1 Knockdown by siRNA. **A** Real-time polymerase chain reaction (RT-PCR) and **B** Western blotting were used to determine PAIP1 expression in the four HCC cell lines relative to that of the normal hepatocyte cell line L02. **C** RT-PCR and **D** Western blotting validated siRNA-mediated PAIP1 knockdown in the SMMC-7721 cell line. The HEPG2 cell line displayed similar siRNA-mediated PAIP1 knockdown (data not shown). GAPDH served as the loading control in all experiments. Results are displayed as means and standard deviations (SDs) from three independent experiments. **P < 0.01

First, PAIP1 mRNA expression levels were analyzed in four human HCC cell lines (SMMC-7721, HEPG2, HUH-7, HEP3B) relative to the normal human hepatocyte cell line L02. PAIP1 transcript levels were upregulated in all four HCC cell lines, and the relative PAIP1 transcript levels were similar in all four HCC cell lines (*P* < 0.01, Fig. 1A). Then, PAIP1 protein expression levels were analyzed in the human HCC cell lines relative to the normal human hepatocyte cell line L02. PAIP1 protein levels were upregulated in all four HCC cell lines, and the relative PAIP1 protein levels were similar in all four HCC cell lines (*P* < 0.01, Fig. 1B).

### Efficient paip1 knockdown by lentivirally-delivered siRNA

The SMMC-7721 and HEPG2 cell lines were infected with lentivirus carrying a PAIP1-siRNA or a negative control-siRNA (NC-siRNA). Lentiviral infection efficiency was assessed by GFP expression 24 h after infection, where we found a lentiviral infection rate exceeding 80% (Additional file 1: Fig. S1). PAIP1 mRNA expression was significantly decreased after siRNA-mediated PAIP1 knockdown (*P* < 0.01, Fig. 1C). Accordingly, Western blotting confirmed that PAIP1 protein expression levels were notably decreased following siRNA-mediated PAIP1 knockdown (Fig. 1D).

### PAIP1 knockdown inhibited HCC cell proliferation

**Fig.2:**
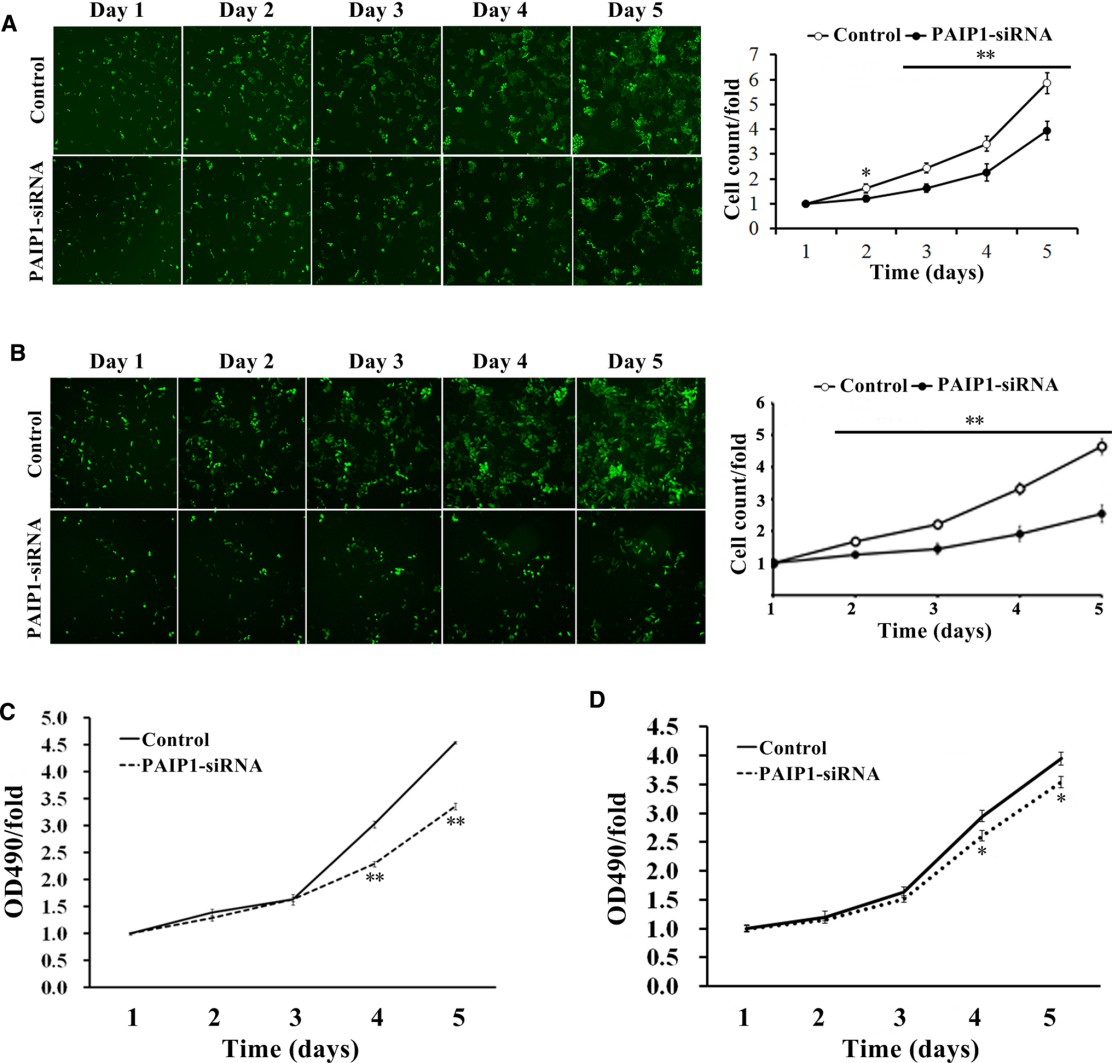
Effect of PAIP1 Knockdown on HCC Cell Proliferation**.** **A**, **B** Cellomics experiments for **A** SMMC-7721 cell proliferation and **B** HEPG2 cell proliferation. Representative images of green fluorescence protein (GFP)-expressing cells over time (left panels, image magnification: 10 ×). Proliferation curves (right panels) were constructed based on GFP expression. The cell count/fold represents the fold-change of the cell count for each respective day normalized to the original cell count on day one. **C**, **D** MTT experiments for **C** SMMC-7721 cell proliferation and **D** HEPG2 cell proliferation. OD490/fold represents the fold-change of the OD490 value for each respective day normalized to the original OD490 value on day one. Results are displayed as means and standard deviations (SDs) from three independent experiments. *P < 0.05, **P < 0.01

In Cellomics assay, PAIP1 knockdown significantly suppressed cell proliferation in SMMC-7721 cells, as demonstrated by decreased GFP + cell counts over a five-day time course (*P* < 0.01, Fig. 2A). Specifically, cell counts decreased by 43% and 46% in the PAIP1-siRNA cells on days four and five, respectively (Fig. 2A). Similarly, PAIP1 knockdown significantly suppressed cell proliferation in HEPG2 cells (*P* < 0.01, Fig. 2B). Specifically, cell counts decreased by 44% and 45% in the PAIP1-siRNA cells on days four and five, respectively (Fig. 2B).

In the MTT assay, PAIP1-siRNA SMMC-7721 cells showed significantly less proliferation over the five-day time course relative to their control counterparts (*P* < 0.01, Fig. 2C). Specifically, cell absorbance decreased by 24% and 26% in the PAIP1-siRNA cells on days four and five, respectively (Fig. 2C). Similarly, PAIP1-siRNA HEPG2 cells showed significantly less proliferation over the five-day time course relative to their control counterparts (*P* < 0.05, Fig. 2D). Specifically, cell absorbance decreased by 11% and 11% in the PAIP1-siRNA cells on days four and five, respectively (Fig. 2D).

### PAIP1 knockdown increased HCC cell apoptosis and reduced colony formation

**Fig. 3:**
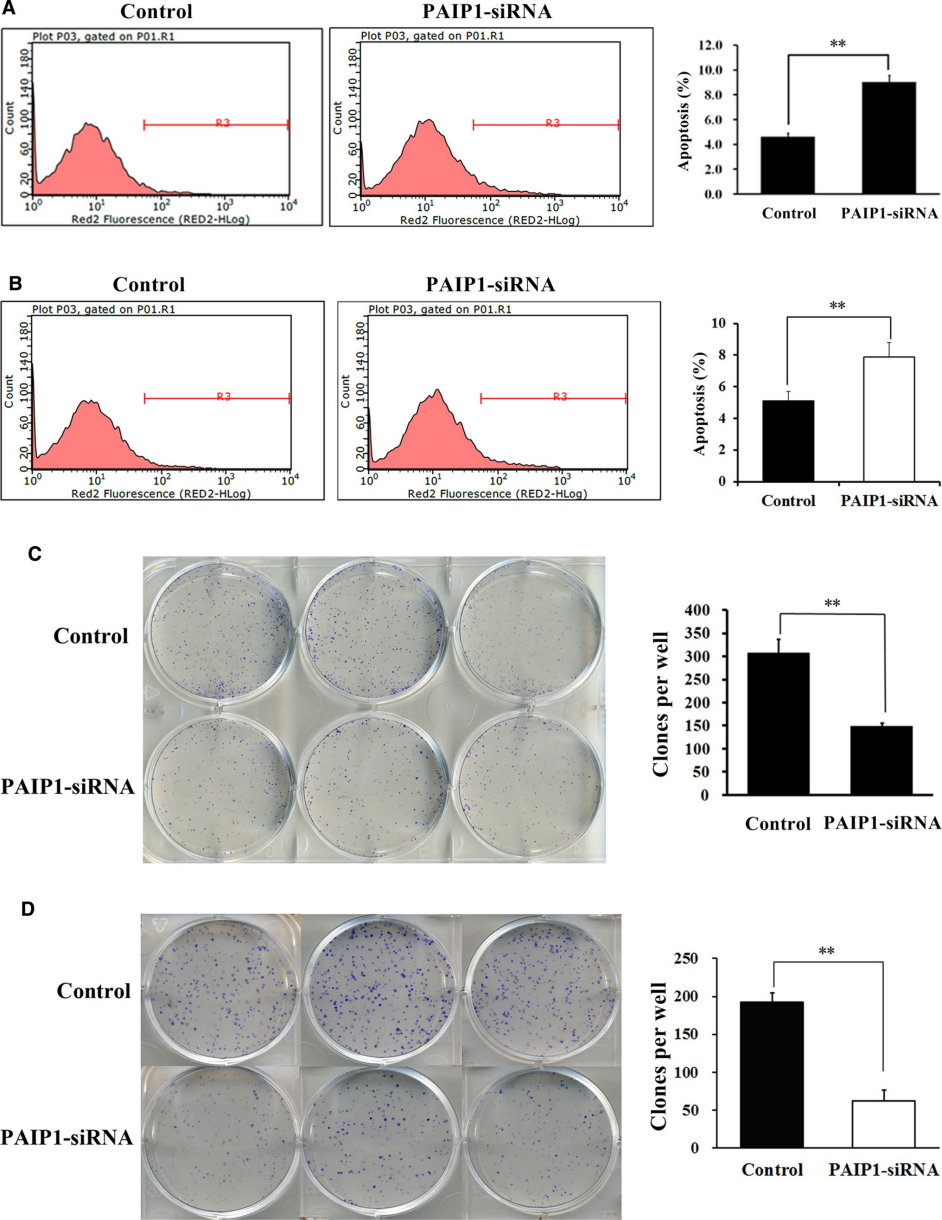
Effect of PAIP1 Knockdown on HCC Cell Apoptosis and Colony Formation**.** **A**, **B** Annexin V/PI-FACS analysis of apoptosis levels in **A** SMMC-7721 and **B** HEPG2 cells. **C**, **D** Assessment of colony formation ability in **C** SMMC-7721 and **D** HEPG2 cells. Cells were plated in a six-well plate (500 cells/well) for a period of 10 days. Clones were counted after staining with Giemsa. Results are displayed as means and standard deviations (SDs) from three independent experiments. *P < 0.05, **P < 0.01

PAIP1 knockdown in SMMC-7221 cells induced a 95% increase in apoptosis rate (*P* < 0.01, Fig. 3A). In addition, PAIP1 knockdown in HEPG2 cells induced a 54% increase in apoptosis rate (*P* < 0.01, Fig. 3B).

Then, PAIP1 knockdown resulted in a 52% decrease in clone formation rates in SMMC-7221 cells (*P* < 0.01, Fig. 3C). In addition, PAIP1 knockdown resulted in a 68% decrease in clone formation rates in HEPG2 cells (*P* < 0.01, Fig. 3D).

### PAIP1 knockdown induced G2/M phase shift in HCC cells

**Fig. 4:**
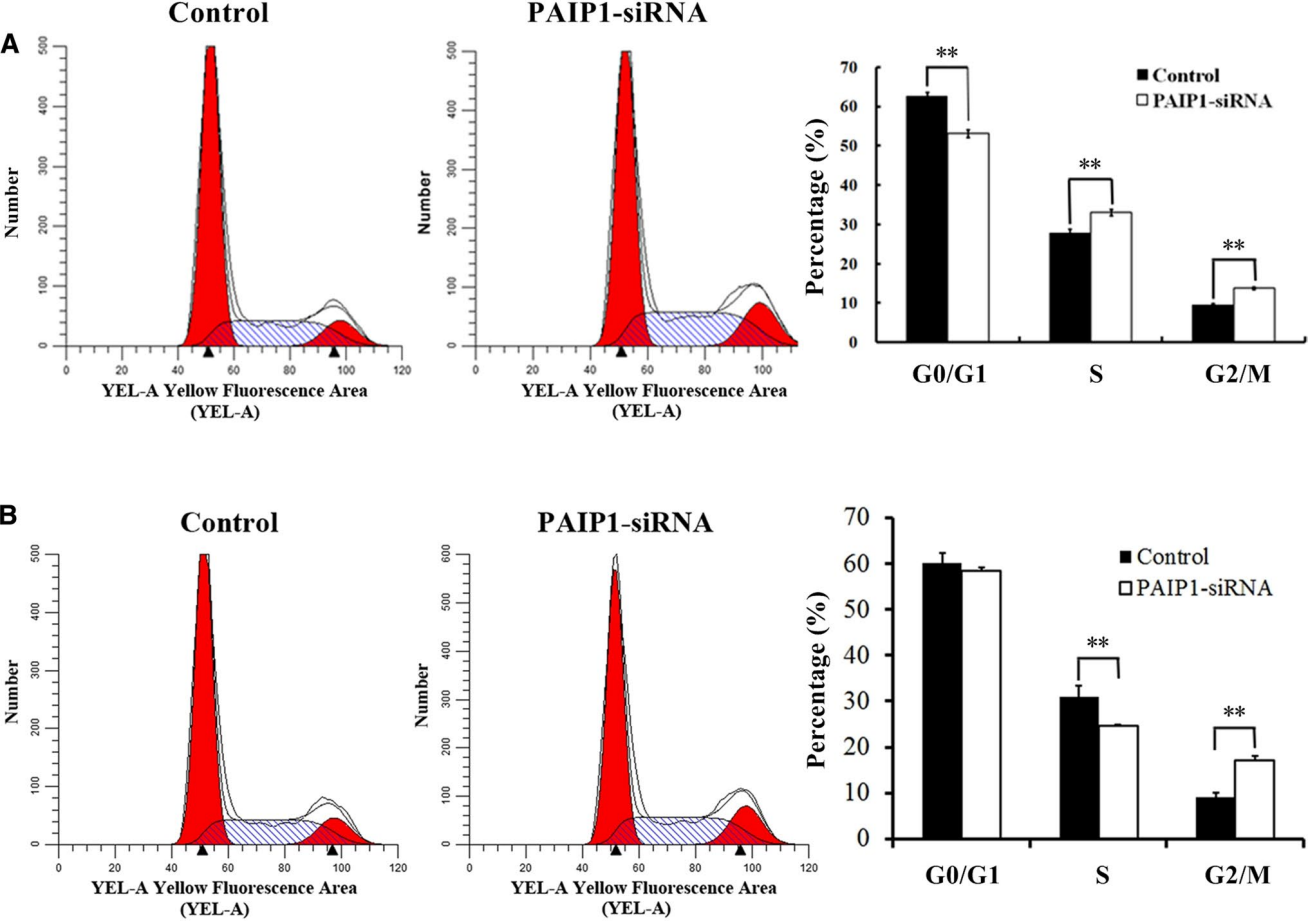
Effect of PAIP1 Knockdown on HCC Cell Cycle Progression**.** PI-FACS analysis of cell cycle progression in **A** SMMC-7721 and **B** HEPG2 cells. Results are displayed as means and standard deviations (SDs) from three independent experiments. *P < 0.05, **P < 0.01

To investigate whether PAIP1 silencing leads to cell cycle defects, SMMC-7721 and HEPG2 cells lentivirally-infected with either PAIP1-siRNA or NC-siRNA were subjected to cell cycle analysis by staining with PI and subsequent FACS analysis. PAIP1 knockdown resulted in a 45% increase in the G2/M phase cell percentage in SMMC-7221 cells (*P* < 0.01, Fig. 4A). In addition, PAIP1 knockdown resulted in a 90% increase in the G2/M phase cell percentage in HEPG2 cells (*P* < 0.01, Fig. 4B).

### PAIP1 knockdown suppressed SMMC-7721 xenograft tumor growth in nude mice

**Fig. 5:**
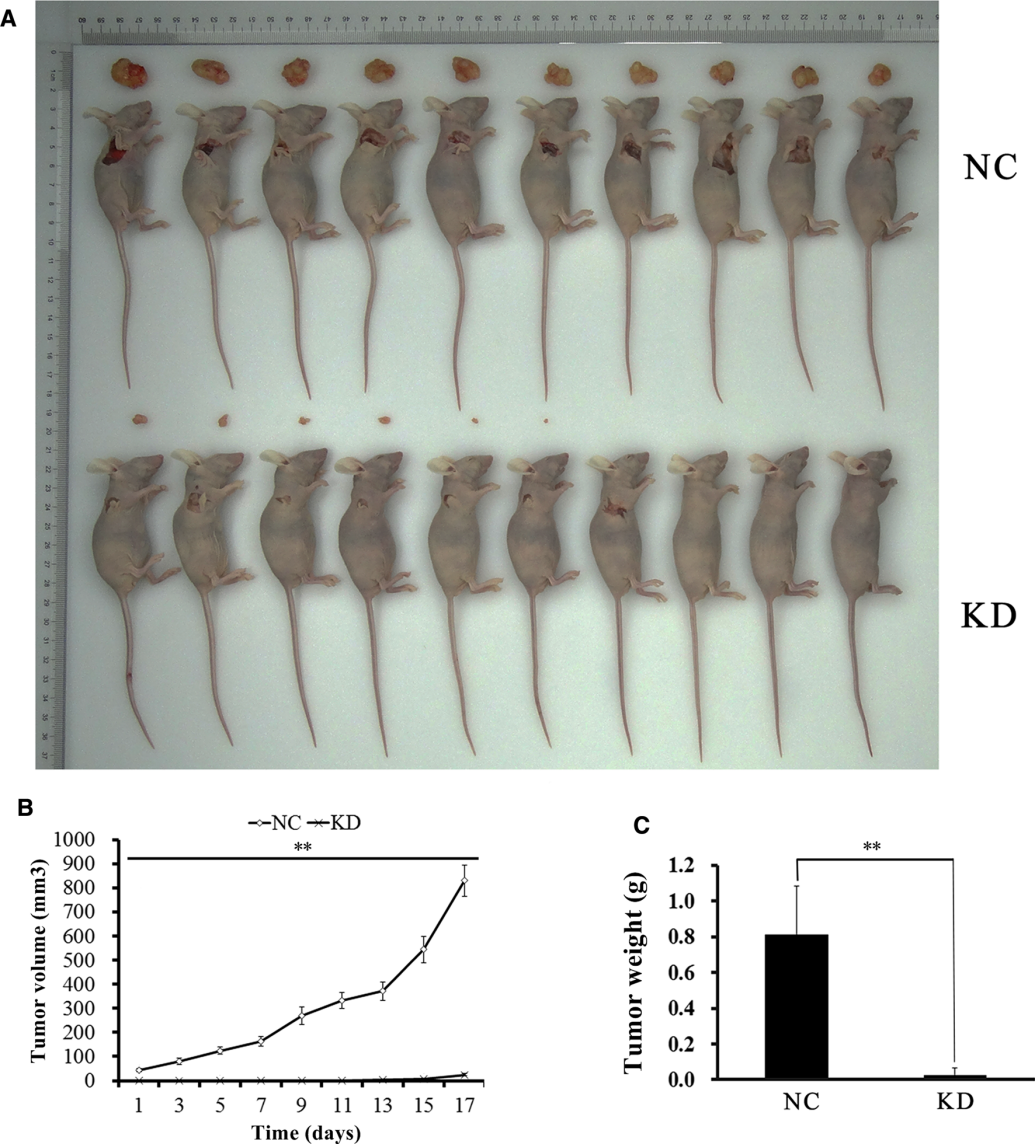
Effect of PAIP1 Knockdown on In Vivo SMMC-7721 Xenograft Tumorigenicity. **A** SMMC-7721 cells were subcutaneously (s.c.) injected into the dorsal flanks of nude mice. **B** Graph of xenograft tumor volumes over the initial 17-day period. **C** Final tumor weights were measured 28 days after s.c. injection. *NC* negative control tumor group, *KD* PAIP1-knockdown tumor group. *P < 0.05, **P < 0.01 means significant difference between NC and KD group from the beginning day

SMMC-7721 cells with/without PAIP1 knockdown were s.c. injected into the dorsal flank of nude mice (Fig. 5A). Remarkably, the average xenograft tumor volume was strikingly reduced by 97% in the KD group on day 17 post-inoculation (*P* < 0.01, Fig. 5B). At 28 days post-inoculation, KD mice carried tumors that were 97% less massive than their NC counterparts (*P* < 0.01, Fig. 5C).

### PAIP1 knockdown induced cyclin D pathway dysregulation

**Fig. 6:**
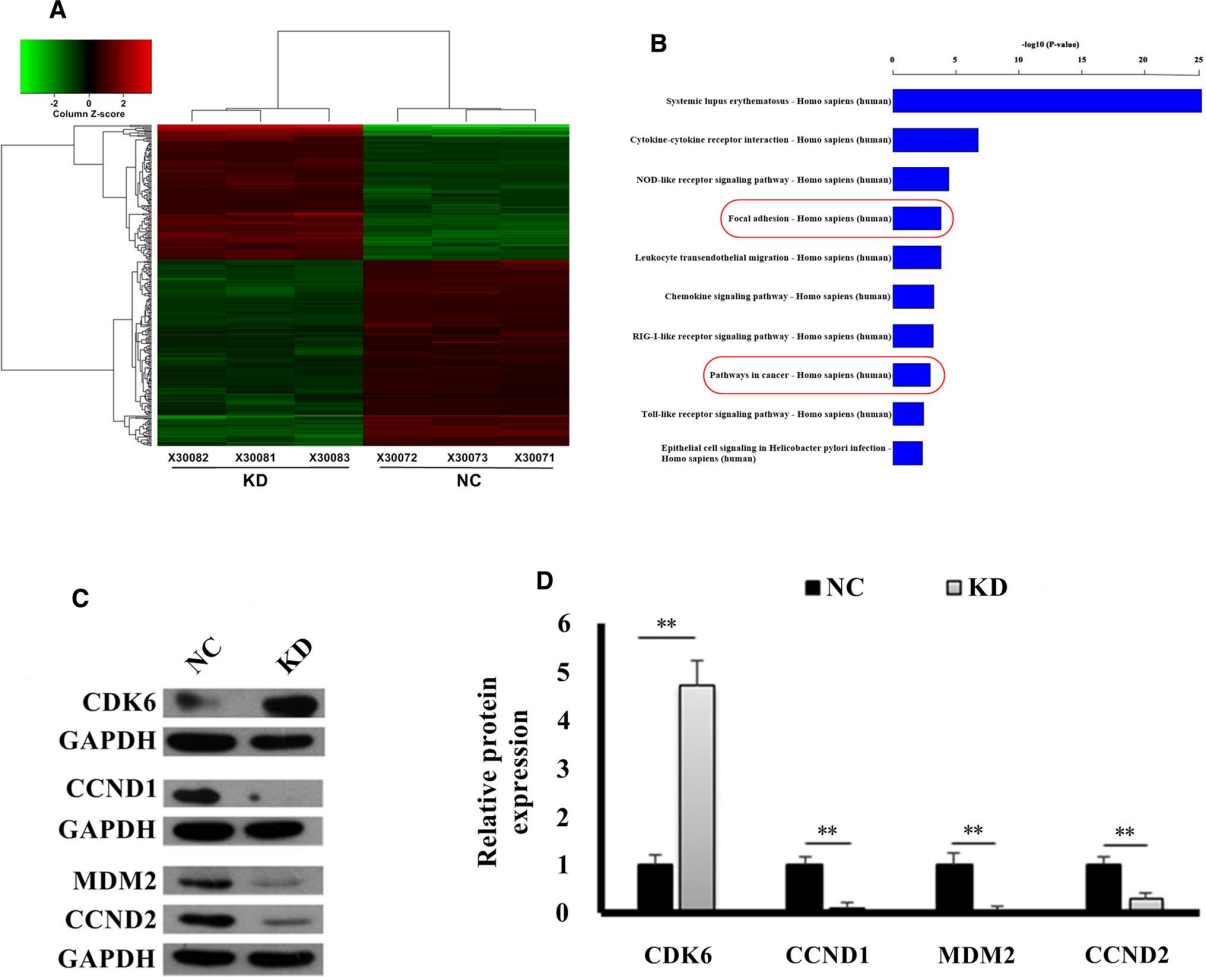
Cyclin D Pathway Dysregulation Induced by PAIP1 Knockdown**.** **A** Cluster analysis of miRNA expression in PAIP1-knockdown (KD) and negative control (NC) SMMC-7721 xenograft tumor cells. Each row represents a unique miRNA, and each column represents an individual tumor sample (n = 3 tumors in each group). Red or green coloring represents elevated or decreased expression, respectively. **B** Results of the KEGG pathway analysis showing the top ten highest-ranking KEGG pathways. The ‘focal adhesion’ and ‘pathways in cancer’ pathways have been circled in red. **C** Representative Western blots of four key cyclin **D** pathway genes. GAPDH served as loading control. **D** The relative expression of each protein is graphically displayed. Results are displayed as means and standard deviations (SDs) from three independent experiments. *P < 0.05, **P < 0.01

miRNAs regulate their target genes by decreasing their mRNA levels [21]. Thus, to better understand the molecular mechanism(s) underlying PAIP1’s effects in HCC cells, we examined the expression of differentially-expressed mature human miRNAs between the NC and KD samples using a miRNA microarray analysis. Cluster analysis was able to clearly distinguish the miRNA microarray profiles of NC and KD tumors (Fig. 6A).

TargetScan analysis was then applied to predict putative target genes for the differentially-expressed mature human miRNAs between the NC and KD tumors. We then analyzed the enrichment of these putative target genes in the KEGG pathway database. The resulting KEGG pathway analysis produced several noteworthy biological pathways that have been previously associated with oncogenesis, including ‘focal adhesion’ and ‘pathways in cancer’ (Fig. 6B) [22–24].

Based upon the TargetScan analysis and the significant dysregulation in the KEGG ‘focal adhesion’ and ‘pathways in cancer’ pathways, we identified the focal adhesion/cancer-associated cyclin D pathway as a putative target pathway of PAIP1 dysregulation in HCC cells [25–27]. On this basis, we sought to validate whether PAIP1 knockdown significantly affects the expression of key cyclin D pathway proteins (i.e., CDK6, CCND1, CCND2, and MDM2). Applying Western blotting analysis on xenograft tumors (Fig. 6C), we demonstrated that the expression of CDK6 – a direct target of one of the differentially-expressed miRNAs – is significantly upregulated in KD tumors (*P* < 0.01, Fig. 6D). On the other hand, we observed significant downregulation in CCND1, CCND2, and MDM2 in KD tumors (*P* < 0.01, Fig. 6D).

## Discussion

Here, we identified PAIP1 as an oncogene in HCC cells and provided a basis for the potential application of PAIP1 in targeted–therapy of cancer. Our initial experiments revealed significantly elevated expression of PAIP1 in four HCC cell lines. Furthermore, our results displayed that PAIP1 knockdown inhibits proliferation, induces apoptosis, reduces colony formation, and promotes G2/M phase shift in these HCC cell lines. But PAIP1 knockdown caused S phase arrest in SMMC7721 cells but not in HepG2 cells. This might be due to the difference between these two cell lines. Cell cycle distribution was not the major target of PAIP1 in HepG2 cells. Furthermore, depletion of PAIP1 expression suppressed SMMC-7721 xenograft tumorigenesis in a nude mouse model. Bioinformatics analysis and immunoblotting on SMMC-7721 xenograft tumor cells revealed that PAIP1 knockdown dysregulates several cyclin D pathway proteins. These findings suggest that PAIP1 may be a critical oncogene in HCC.

MiRNA microarray was a common method to explore the mechanism in cancer. mRNAs are generally the target of miRNAs, so miRNA array could reflect the expression pattern of mRNAs. Even, miRNA assay might give us more information than mRNA array. Although more research is required to reveal the underlying mechanism of PAIP1 in tumorigenesis, there is a substantial body of evidence linking dysregulations in PAIP1-associated translational initiation factors with oncogenesis [28]. Kratze et al. has reported that many malignancies display hyper-activation of eIF4 proteins (i.e., eIF4A, eIF4E, eIF4G, and 4E-BP proteins), resulting in enhanced binding to the 5’ cap and preferential translation of cancer-associated genes, including cyclin D1, c-myc, and ornithine decarboxylase [29]. As PAIP1 stabilizes PABP’s interaction with eIF4G [6], the PAIP1 upregulation in HCC cells observed here may promote the translation of oncogenic mRNAs by promoting eIF4G hyper-activation. In miRNA microarray assay, we identified the cyclin D pathway as a putative target pathway of PAIP1 dysregulation in HCC cells [25–27]. We then demonstrated that PAIP1 significantly affects the expression of four cyclin D pathway genes (namely, CDK6, CCND1, CCND2, and MDM2).

Most notably, we found that CCND1 and CCND2 were significantly downregulated upon PAIP1 knockdown in SMMC-7721 xenograft tumor cells, suggesting that PAIP1 promotes CCND1, CCND2, and MDM2 expression in HCC cells. The cyclin D proteins CCND1 and CCND2 have been positively linked to HCC oncogenesis [30–32]. Mechanistically, CCND1 and CCND2 interact with CDK6 and CDK4, forming the cyclin D/CDK6 and cyclin D/CDK4 complexes. These complexes hyper-phosphorylate retinoblastoma (Rb), thereby facilitating the G1-S phase transition [33]. Thus, many cancer cells depend on elevated cyclin D/CDK activity to transition through this regulatory checkpoint [33]. Consistently,l PAIP1 knockdown downregulates CCND1 and CCND2 expression in HCC cells, thereby restricting cyclin D/CDK activity and producing the observed anti-malignant effects. Based on Kratze et al.’s proposed model [29], we speculate that PAIP1 expression in HCC cells promotes eIF4G hyper-activation, thereby promoting preferential translation of CCND1 and CCND2 transcripts.

Interestingly, we also found that CDK6 was significantly upregulated upon PAIP1 knockdown in SMMC-7721 xenograft tumor cells, suggesting that PAIP1 negatively regulates CDK6 expression in HCC cells. In this regard, PAIP1 surprisingly behaves as a tumor suppressor. PAIP1’s target PABP has been shown to promote the formation and/or stabilization of nuclear TAR-DNA binding protein of 43 kDa (TDP-43), a negative regulator of CDK6 and its downstream targets Rb and Rb2/p130 [34, 35]. Therefore, PAIP1 may promote PABP’s interaction with TDP-43, thereby negatively regulating the oncogenic CDK6-Rb pathway.

Then, we found that MDM2 was significantly downregulated upon PAIP1 knockdown in SMMC-7721 xenograft tumor cells, suggesting that PAIP1 promotes MDM2 expression in HCC cells. MDM2 functions as a negative regulator of the tumor suppressor p53 and may be responsible for inactivation of p53 in HCC tumors [36]. Mechanistically, MDM2 catalyzes p53 ubiquitination, targeting the p53 protein for degradation [37]. Normal p53 activity reroutes cells that have undergone oncogenic damage to G1 phase arrest via inhibiting cyclin D/CDK4 [37]. Disruption of p53 activity by MDM2 eliminates p53-mediated inhibition of cyclin D/CDK4, enabling oncogenes to drive uncontrolled cell proliferation [37]. Consistently, PAIP1 knockdown downregulates MDM2 expression in HCC cells, thereby maintaining p53 activity and producing the observed anti-malignant effects. However, much more experiments are necessary to further support the foundation in this study. For example, in miRNA microarray assay, cytokine-cytokine receptor interaction was another signaling pathway dominantly affected by PAIP1. The interaction of cytokines with their receptor are important in processes such as s cell proliferation, migration, EMT, and so on. In addition, the data suggested that PAIP1 was an oncogene in liver cancer. Then, if PAIP1 could be considered as a potential drug target? What’s role of PAIP1 in chemotherapy of patients with liver cancer? These questions will be a critical issue in our following study.

In summary, the present study showed that PAIP1 plays an oncogene role in the tumorigenesis of HCC, and its oncogenic effects may interact with cyclin D signaling pathway. Our results provide informational support for using PAIP1 as potential therapeutic target for HCC therapy.

## Supplementary Information

**Additional file 1: Fig. S1.** Lentiviral Infection Efficiency in SMMC-7721 Cells.

## Data Availability

The data used to support the findings of this study are available from the corresponding author upon request.
