# Supplementary material for: PAIP1 is a novel oncogene in human hepatocellular carcinoma
Source: Discov Oncol. 2022 Nov 27;13:132. doi: 10.1007/s12672-022-00530-0 (PMC9702235; doi:10.1007/s12672-022-00530-0)

**Supplementary Fig.1** Lentiviral Infection Efficiency in SMMC-7721 Cells


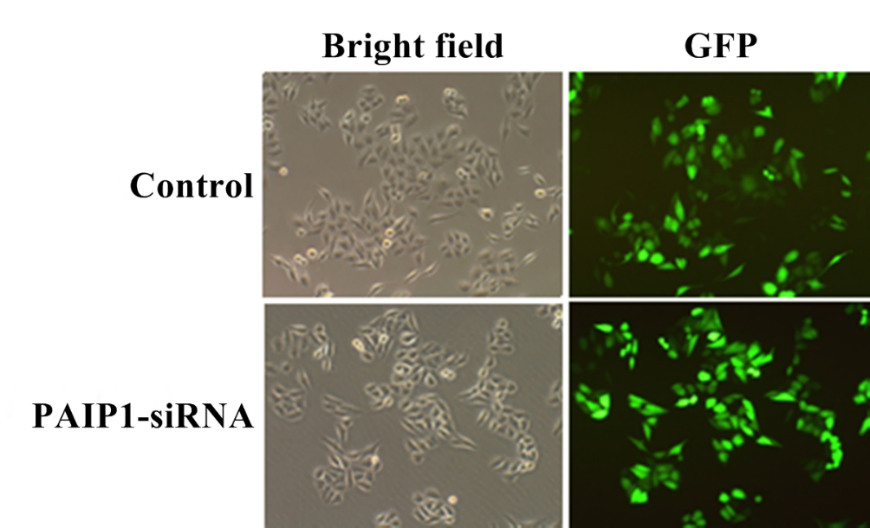


Representative bright field and green fluorescent protein (GFP) microscopic images of SMMC-7721 cells 24 hours after lentiviral infection containing the negative control-siRNA (Control) or the PAIP1-siRNA (magnification: 10×). More than 80% of cells in each experimental group displayed positive GFP expression, indicating a strong lentiviral infection efficiency. HEPG2 cells displayed a similar lentiviral infection efficiency to SMMC-7721 cells (images not shown).

Figure S1. The knockdown efficiency of PAIP1 in HepG2 cells.

The original gel images:

Figure 1 D: PAIP1 and GAPDH


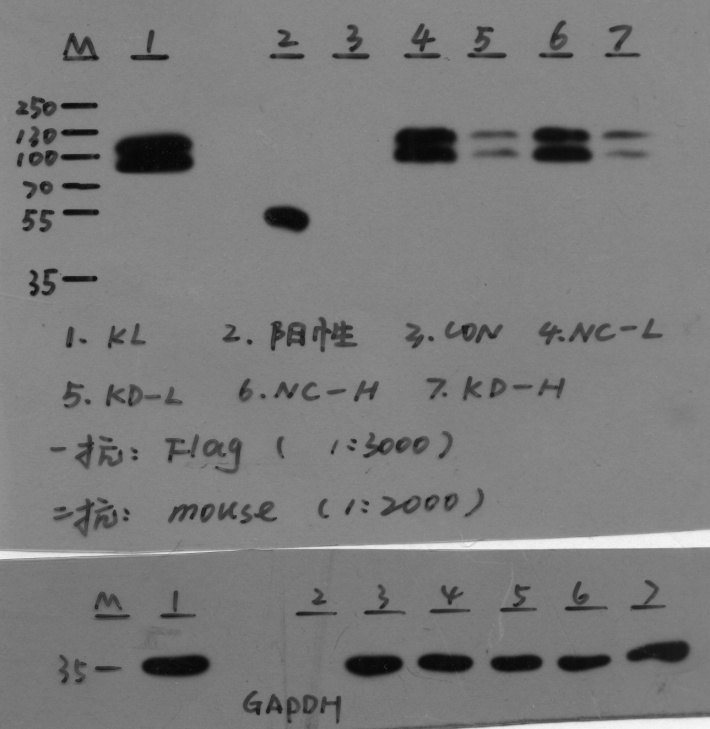


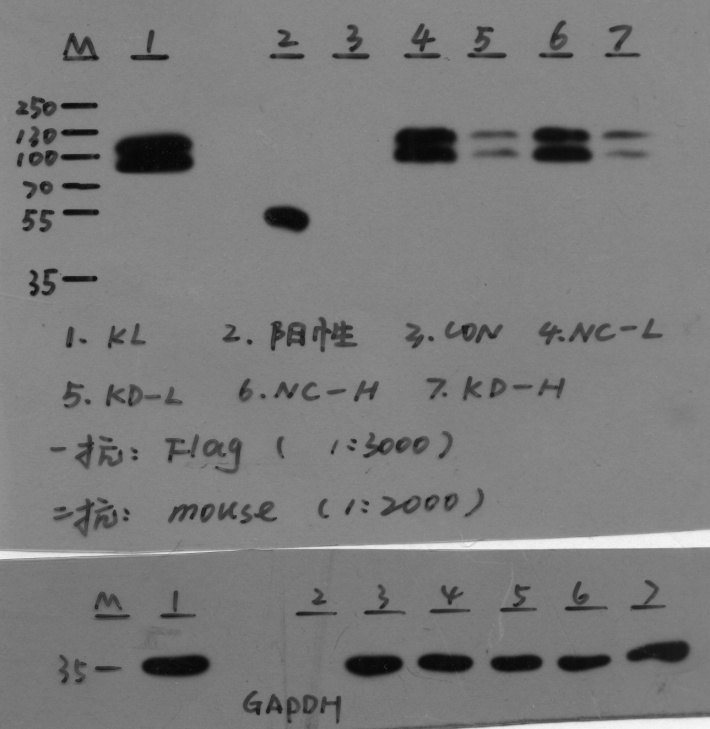


Figure 6C:

CDK6 and GAPDH


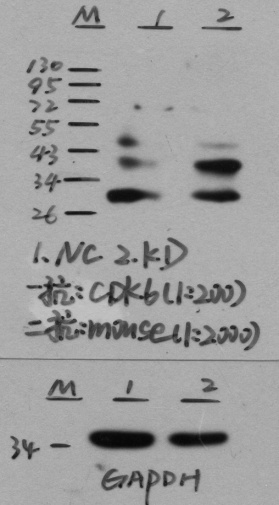


CCND1 and GAPDH


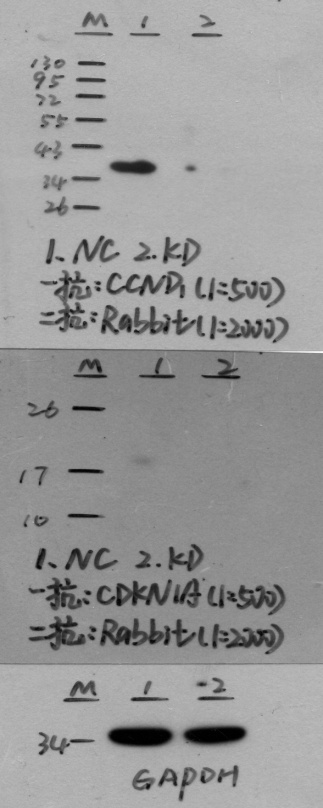


MDM2, CCND2 and GAPDH


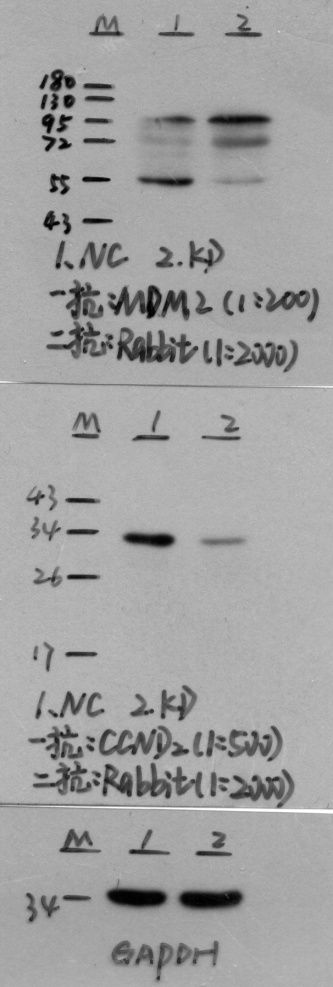

Supplement: Supplementary file 1 — Additional file 1: Fig. S1. Lentiviral Infection Efficiency in SMMC-7721 Cells. [file 12672_2022_530_MOESM1_ESM.docx]
